# From information to action― a phase-based analysis of patient engagement in inpatient fall prevention: A scoping review

**DOI:** 10.1016/j.ijnsa.2026.100677

**Published:** 2026-09-10

**Authors:** Sayaka Nishihara, Masae Satoh, Yuriko Iijima, Mika Katoh, Hiromi Shimmura, Rika Kawahara, Natsuko Shimazawa

**Affiliations:** Department of Nursing, Graduate School of Medicine, Yokohama City University, 3-9 Fukuura, Kanazawa-ku, Yokohama, 236-0004, Japan

**Keywords:** Accidental falls, Fall prevention, Patient participation, Engagement, Involvement, Activation, Empowerment

## Abstract

**Background:**

Inpatient falls are a major patient safety concern worldwide. Although patient engagement has been increasingly emphasised in fall prevention, its distribution and depth across the prevention process remain poorly understood.

**Objective:**

This scoping review examined how patient engagement is structured across the phases of inpatient fall prevention worldwide, focusing on decisional ownership and the contextual and structural factors influencing its implementation.

**Design:**

A scoping review was conducted in accordance with the Joanna Briggs Institute methodology.

**Methods:**

PubMed, Web of Science, and CINAHL were searched for English-language studies published between January 2015 and July 2026. The final search was conducted on 16 July 2026. Patient engagement was conceptualised as decisional ownership and classified into Level 1 (passive engagement), Level 2 (shared decision-making), and Level 3 (autonomous decision-making). The inpatient fall prevention process was organised into four phases: risk awareness, co-planning, goal setting, and self-management. Data were synthesised descriptively, incorporating cognitive requirements, family involvement, and information-sharing approaches.

**Results:**

Patient engagement varied across the four phases of inpatient fall prevention and was unevenly distributed throughout the prevention process. Engagement was concentrated in the risk awareness and co-planning phases, where Level 2 (shared decision-making) was occasionally achieved. In contrast, goal setting was often implicit or absent, and patient involvement in self-management remained limited. The extent of engagement was influenced by patients’ cognitive capacity, with family involvement serving as a proxy in some interventions, although this role was inconsistently translated into active participation or behavioural change.

**Conclusion:**

Patient engagement in inpatient fall prevention does not extend uniformly across the care process. Current interventions primarily support shared decision-making but rarely facilitate autonomous patient involvement. Future interventions should incorporate explicit goal setting, strengthen self-management, integrate proxy engagement for patients with cognitive impairment, and align information sharing with decision-making support. This review reconceptualises patient engagement as a phase-dependent, structurally mediated process and identifies important gaps in the design of current interventions.

**What is already known**

• Inpatient falls are a major patient safety concern, and patient engagement is widely promoted as an important component of fall prevention.
• Existing patient engagement interventions improve patients' knowledge and awareness of fall prevention; however, how engagement is structured across the care processes remains unclear.

**What this paper adds**

• This review introduces a phase-based analytical framework that conceptualises patient engagement as decisional ownership across the inpatient fall prevention process.
• It demonstrates that current interventions largely support information sharing and shared decision-making but seldom facilitate progression to explicit goal setting and patient self-management.
• These findings provide a conceptual basis for designing future patient engagement interventions that bridge the gap between information provision and meaningful preventive action.

## 1 Background

Inpatient falls remain one of the most common adverse events in hospitals worldwide and continue to represent a major patient safety challenge (McKercher et al., 2024; Morris, 2025). Falls are associated with fractures, functional decline, prolonged hospitalisation, increased healthcare costs, and reduced quality of life (Dykes et al., 2023; Takase, 2023; Hirose et al., 2018). Beyond their adverse consequences for patients, inpatient falls are increasingly recognised as indicators of hospital safety performance and healthcare quality (Bernet et al., 2022; Satoh et al., 2026). In Japan, the nationwide Quality Indicator Project reported an inpatient fall rate of approximately 2.6 falls per 1000 patient-days among hospitals with 600 or more beds (Japan Council for Quality Health Care, 2025), highlighting fall prevention as an ongoing national patient safety priority.

### 1.1 Patient engagement in inpatient fall prevention

Traditional fall-prevention programmes have primarily focused on professional risk assessment, environmental modification, and patient education. Although these interventions improve patients' knowledge and awareness of fall risk, many patients continue to engage in behaviours that increase their likelihood of falling. Recent evidence suggests that knowledge alone is insufficient to promote protective behaviour. (Zhao et al., 2026) demonstrated that substantial gaps remain between fall-prevention education, patients' understanding, and the implementation of recommended preventive behaviours. Likewise, (Takase, 2026), using the revised Protection Motivation Theory, reported that adherence to nurses' fall-prevention recommendations depends not only on receiving information but also on patients' perceptions of risk, confidence in performing preventive behaviours, beliefs regarding intervention effectiveness, and supportive relationships with healthcare professionals. Collectively, these findings indicate that the persistent gap between information and action cannot be explained solely by inadequate patient education.

Recognition of these limitations has led to increasing emphasis on patient engagement as a central component of patient safety. Patient engagement encompasses information exchange, participation in care, shared decision-making, partnership with healthcare professionals, and self-management (Carman et al., 2013; Castro et al., 2016). Shared decision-making further requires patients and healthcare professionals to exchange information, deliberate on available options, and reach mutually agreed decisions (Charles et al., 1997; Elwyn et al., 2012). Importantly, patient engagement is increasingly recognised as a dynamic process rather than a single intervention component, with the degree and nature of participation varying according to the clinical context, the patient's capabilities and preferences, and the support provided by healthcare professionals. Within inpatient fall prevention, this perspective shifts patients from passive recipients of professional advice to active partners who recognise personal risks, contribute to prevention planning, establish meaningful goals, and participate in implementing preventive strategies.

Several interventions have attempted to operationalise patient engagement within hospital fall prevention. The Fall TIPS programme provides individualised bedside communication tools to facilitate collaborative fall-prevention planning among patients, families, and healthcare professionals (Dykes et al., 2017; 2019; 2020; Christiansen et al., 2020), while the Safe Recovery programme combines patient education with behavioural change strategies to encourage active patient participation (Hill et al., 2015). Other interventions have incorporated structured risk-assessment tools, personalised prevention plans, audiovisual education, and patient-centred communication (Guo et al., 2023; Morris et al., 2024; Radecki et al., 2020). More recently, self-efficacy-based interventions, technology-assisted visual feedback, and personalised fall-prevention programmes have further expanded opportunities for patient participation (Tan et al., 2025; Steinmetz et al., 2025; Li et al., 2025; Sultana et al., 2025). These developments reflect a gradual transition from simply informing patients about fall risk towards supporting their active involvement in preventive decision-making and behaviour.

However, patient engagement alone does not necessarily translate into sustained preventive behaviour. Recent studies have shown that patients' participation is influenced by multiple interacting factors, including illness severity, cognitive capacity, perceived fall risk, communication with healthcare professionals, organisational context, and environmental constraints (Mou et al., 2026). Qualitative studies further demonstrate that patients at high risk of falling often perceive preventive measures differently from healthcare professionals and may regard them as restrictive or insufficiently individualised (Yasan et al., 2025; Isaac et al., 2026). Furthermore, implementation studies have reported considerable variation in the adoption of recommended fall-prevention practices across hospitals, reflecting organisational and contextual influences beyond individual patient participation (Alvarado et al., 2025). Together, these findings suggest that patient engagement should be understood as a dynamic, context-dependent process that evolves throughout the course of care rather than as a simple indicator of whether patients participate or not. However, opportunities for patient engagement may vary according to patients' capacity to participate. Patients with cognitive impairment, severe illness, or communication difficulties may face barriers to understanding fall risks, expressing preferences, or engaging in preventive actions. In such circumstances, engagement may require support from healthcare professionals, family members, or caregivers and should therefore be understood in relation to both patient capacity and the context in which participation occurs.

Existing reviews and intervention studies have primarily evaluated whether patient engagement interventions improve knowledge, adherence, or fall-related outcomes. Although these studies provide valuable evidence regarding intervention effectiveness, they offer limited insight into how patient engagement is organised across successive phases of inpatient fall prevention. In particular, it remains unclear when patients primarily receive information, when they participate in shared decision-making, and when they assume greater ownership of preventive actions. Examining patient engagement from the perspective of decisional ownership across the fall-prevention pathway may therefore reveal important structural gaps that remain hidden when engagement is treated as a single, homogeneous construct.

### 1.2 Aim

The aims of this scoping review were to 1) map the characteristics and extent of patient engagement in inpatient fall-prevention interventions worldwide; 2) examine patient engagement across the four phases of inpatient fall prevention (risk awareness, co-planning, goal setting, and self-management) and classify the level of engagement according to patients' decisional ownership; and 3) explore the roles of cognitive requirements, family involvement, and information-sharing processes in supporting the transition from information provision to meaningful preventive action.

## 2 Methods

### 2.1 Study design

This scoping review was conducted in accordance with the Joanna Briggs Institute methodology for evidence synthesis (Pollock et al., 2024) and is reported following the Preferred Reporting Items for Systematic Reviews and Meta-Analyses extension for Scoping Reviews guidelines (Tricco et al., 2018) (Supplementary File 1).

A scoping review methodology was selected because it enables the systematic mapping of heterogeneous evidence, including diverse intervention approaches, forms of patient engagement, and conceptual frameworks. This approach was considered appropriate for examining the characteristics and conceptual structure of patient engagement in inpatient fall prevention.

### 2.2 Eligibility criteria (PCC framework)

This review was guided by the Population–Concept–Context (PCC) framework (Pollock et al., 2024):

• Population: Hospitalised patients
• Concept: Patient engagement in fall prevention
• Context: Hospital setting, including acute and rehabilitation care

Studies were included if they: (a) involved hospitalised patients; (b) examined fall prevention interventions; (c) included elements of patient engagement; and (d) were original research articles published in English.

Studies were excluded if they: (1) focused exclusively on paediatric or neonatal populations; (2) were review articles or study protocols; (3) included only patient education without decision-making or behavioural involvement; (4) were conducted in outpatient or community settings; or (5) focused solely on tool development or conceptual discussions without intervention evaluation.

### 2.3 Conceptual framework and definition of patient engagement

Patient engagement encompasses multiple dimensions, including information exchange, participation, partnership, shared decision-making, and self-management. For the purpose of this review, the level of patient engagement was operationalized in terms of decisional ownership, defined as the extent to which patients had an active role in making or shaping decisions regarding their fall-prevention care. Decisional ownership was selected as the analytical dimension because it provides a common basis for examining the extent of patient involvement across the different phases of fall prevention, including risk awareness, co-planning, goal setting, and self-management. This approach was informed by theoretical frameworks describing varying levels of patient involvement in healthcare decision-making (Elwyn et al., 2012; Carman et al., 2013). Based on this concept, engagement was classified into three levels:

• Level 1 (passive engagement): Decision-making is clinician-led, and patients receive and accept information.
• Level 2 (shared decision-making): Patients participate in decision-making through interactions with healthcare professionals.
• Level 3 (autonomous decision-making): Patients take a leading role in decision-making and behavioural adjustment.

Engagement levels were assigned according to the degree of decisional authority within each phase. Studies were classified as Level 1 when patients only received or confirmed information, Level 2 when they actively participated in decision-making, and Level 3 when they assumed primary responsibility for decision-making and self-regulation.

To examine the distribution of patient engagement across the inpatient fall prevention process, interventions were categorised into four sequential phases: (1) risk awareness/risk assessment, (2) co-planning, (3) goal setting, and (4) self-management. This phase-based framework integrates the general clinical care process with behavioural change theories describing progression from knowledge acquisition to autonomous action (Bandura, 1997; Hibbard et al., 2004). Evaluation was considered an iterative component of goal setting and self-management rather than a separate phase. Engagement levels were assessed independently within each phase, recognising that patient engagement may vary across the care process rather than remain fixed.

Patient activation was used to interpret patients' readiness and willingness to participate in fall-prevention activities (Greene and Hibbard, 2012), whereas self-efficacy was used to interpret patients' confidence in performing preventive behaviours (Bandura, 1997). Together, these concepts helped explain how interventions supported progression from information receipt to active participation and self-management. However, they were used solely as interpretive frameworks and not as classification criteria.

### 2.4 Search strategy

The search strategy was developed and refined in consultation with an experienced medical librarian with expertise in systematic review searching to ensure its comprehensiveness and reproducibility. Searches was conducted in PubMed, Web of Science Core Collection, and CINAHL. The search strategy combined controlled vocabulary and free-text terms related to patient engagement, inpatient falls, and hospital settings. Search terms included synonyms for patient participation (e.g., patient engagement, patient participation, shared decision-making, self-management), falls (e.g., falls, accidental falls), and hospitalised adults. The initial search covered studies published from January 2015 to December 2024. Then, the search was updated using the same search strategy on 16 July 2026 to identify newly published studies. No restrictions other than language (English) and publication type (peer-reviewed original research articles) were applied. The complete search strategies for each database are provided in Supplementary Table S1.

### 2.5 Study selection

**Fig. 1:**
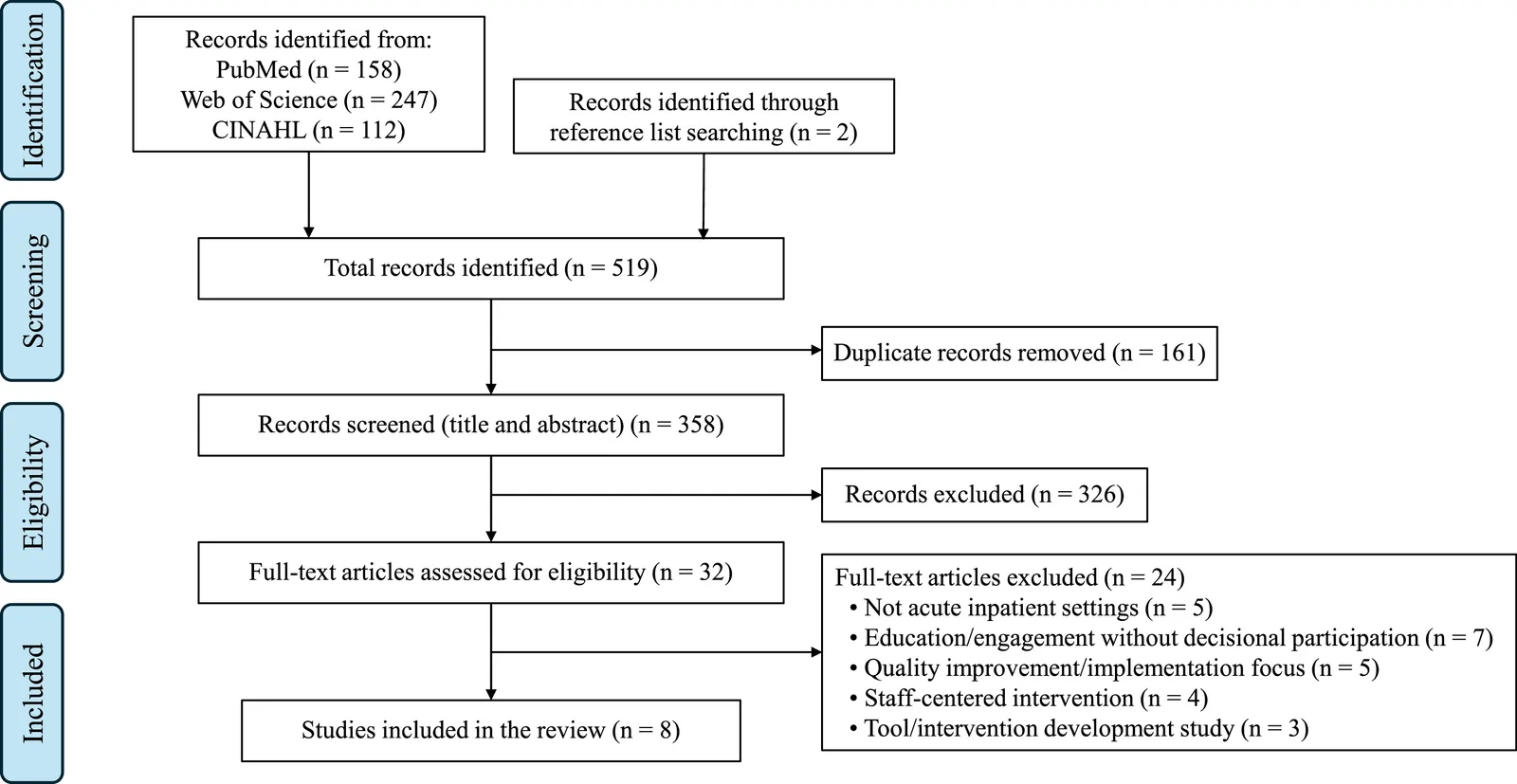
Flow diagram of the study selection process.

Two reviewers (the first and fourth authors) independently screened the titles, abstracts, and full texts of potentially eligible studies. Any disagreements were resolved through discussion until consensus was reached. The selection process is illustrated in a flow diagram (Fig. 1).

### 2.6 Data extraction

Data were extracted using a standardised form developed in advance (Pollock et al., 2024). Extracted variables included study characteristics (author, year, country, design, and sample size), intervention components and delivery methods, risk assessment approaches, presence and modification of care plans, goal setting (explicit, implicit, or not described), self-management elements, family involvement, cognitive eligibility criteria, outcome measures (clinical and patient-reported outcomes), timing and structure of evaluation, and information-sharing modalities. In addition, information relevant to each of the four predefined phases of inpatient fall prevention and the corresponding level of patient engagement was extracted according to the analytical framework described above. Three reviewers (the first, second and fourth authors) independently extracted the data. Any discrepancies were discussed and resolved by mutual agreement.

### 2.7 Data synthesis

A descriptive synthesis approach was used in accordance with the guidance of (Popay et al., 2006). Data extracted from the included studies were synthesised using the predefined analytical framework developed for this review. Verbatim descriptions of intervention content, patient activities, educational components, implementation processes, and reported outcomes were examined and mapped to the four predefined phases of inpatient fall prevention (risk awareness/risk assessment, co-planning, goal setting, and self-management). The data synthesis and analysis were conducted by two reviewers (the first and second authors), with disagreements resolved through discussion.

Within each phase, the corresponding level of patient engagement was classified as passive engagement, shared decision-making, or autonomous decision-making according to the decisional ownership framework. The concepts of patient activation and self-efficacy were then used as interpretive frameworks to examine how interventions supported patients in progressing from information receipt to active participation and self-management. These concepts informed the interpretation of patient engagement across the included studies but were not used as classification criteria.

Finally, intervention characteristics and reported findings were compared descriptively across studies to identify patterns, similarities, and differences relevant to each review question.

### 2.8 Risk of bias assessment

As this was a scoping review, a formal risk of bias assessment was not performed in line with methodological guidance (Munn et al., 2018; Pollock et al., 2024). The purpose of the review was to map and structurally analyse the existing evidence on patient engagement in inpatient fall prevention rather than to evaluate methodological quality or estimate intervention effects.

## 3 Results

### 3.1 Characteristics of the included studies

A total of 519 records were identified through database searching. After removing 161 duplicates, 358 records were screened based on titles and abstracts. Of these, 326 records were excluded for not meeting the inclusion criteria. The remaining 32 full-text articles were retrieved and assessed for eligibility, and eight studies were ultimately included in this review (Fig. 1; Table 1). Of these, five were conducted in the United States, two in Australia, and one in China. No studies from Europe or Japan met the inclusion criteria. Study designs included randomised controlled trials, non-randomised intervention studies, and observational or implementation studies. All studies involved hospitalised patients: six were conducted in acute care settings and two in rehabilitation or geriatric wards. Sample sizes ranged from 116 to 37,231 participants. The included interventions were categorised into three broad types: (1) Fall TIPS-based programmes focusing on standardised risk assessment and bedside information sharing; (2) education and behaviour-change interventions, including the Safe Recovery programme and allied health assistant-delivered education; and (3) engagement-oriented interventions incorporating patient self-assessment or structured engagement frameworks, such as technology-based programmes and the Patient Fall Self-Assessment Tool (Table 2). Although all interventions aimed to promote patient engagement in fall prevention, they differed substantially in their design, implementation, and intended level of patient engagement.

**Table 1:** Characteristics of Included Studies and Interventions.

Author (year) & Country	Study design	Setting	Sample size	Intervention (name/type)	Clinical outcomes	Patient-reported and process-related measures	Cognitive eligibility
Dykes (2017) USA	Pilot pre–post study	Multiple acute care wards (e.g. oncology, neurology, internal medicine)	Not explicitly reported	Fall TIPS programme	Falls, injurious falls	Patient awareness of fall risk and prevention plan; adherence to the prevention plan*	Not reported
Duckworth (2019) USA	Implementation study	Neurology and medical wards (three acute care hospitals)	1209 engagement audits	Fall TIPS programme (display modality comparison: EHR-linked, laminated poster, bedside display)	—	Patient/family understanding of fall risk and prevention plan; program adherence (audit-based)*	Included; proxy allowed
Christiansen (2020) USA	Multisite observational study	Medical units (three acute care hospitals)	343 patients (pre 158 / post 185)	Fall TIPS program (PAM-13 evaluation study)	—	Patient activation (PAM-13); program adherence (patients, nurses)*	Not restricted (measurement restricted)
Dykes (2020) USA	Stepped-wedge cluster non-randomised trial	Not specified (acute care hospital wards)	37,231 patients (pre 17,948 / post 19,283)	Intervention: Fall TIPS program Control: Usual care	Falls, injurious falls	Program adherence* (patients, nurses)	Included; proxy implied
Hill (2015) Australia	Stepped-wedge cluster RCT	Rehabilitation units	3606 patients	Intervention: Safe Recovery program Control: Usual care	Falls, injurious falls, length of stay	Delivery of education; completion of action plans	Restricted (cognitively intact required)
Morris (2024) Australia	Feasibility randomised controlled trial	Geriatric and rehabilitation wards	541 patients (270 control / 271 intervention); 12 allied health assistants	Allied health assistant-delivered patient falls prevention education (scripted conversation + pamphlet)	Falls; injurious falls; falls requiring transfer to acute medical facility	Feasibility*; fidelity*; patient knowledge; workforce acceptability	Restricted (cognitive impairment excluded)
Guo (2023) China	Quasi-experimental study	Multiple acute care wards (e.g. oncology, cardiology, neurology)	116 patients (58 intervention / 58 control)	Intervention: Fall prevention program based on a patient engagement framework Control: Guideline-based manual implementation	Falls	Knowledge–attitude–practice (KAP); fall-related self-efficacy (MFES)	Restricted (no cognitive impairment)
Radecki (2020) USA	Quality improvement study	Surgical wards (general surgery, cardiovascular surgery, oncology)	203 patients (103 baseline / 100 intervention) + 40 nurses	Patient Fall Self-Assessment Tool (PFAT)	Falls, injurious falls	Patient knowledge and fall-prevention behaviours; prevention plan adherence (PFAT)*; nurse usability	Restricted (no cognitive deficit)

EHR, electronic health record; Fall TIPS, Tailoring Interventions for Patient Safety; PAM-13, 13-item Patient Activation Measure; PFAT, Patient Fall Self-Assessment Tool; KAP, Knowledge, Attitudes, and Practices; MFES, Modified Falls Efficacy Scale.

* Measures marked with an asterisk represent implementation or process-related measures (e.g., adherence, feasibility, or fidelity) that were also reported as study outcomes in the original publications.

**Table 2:** Program components and methods of the included interventions.

Author (year)	Intervention description
Dykes (2017)	Fall TIPS programme: Nurse-led fall risk assessment using the Morse Fall Scale; individualised prevention plans linked to evidence-based interventions and displayed at bedside for review with patients
Duckworth (2019)	Fall TIPS (modality comparison): Nurse-led Morse Fall Scale assessment with EHR-linked CDS; tailored plans delivered via multiple display modalities and monitored for adherence
Christiansen (2020)	Fall TIPS programme with EHR-integrated CDS; individualised plans reviewed with patients; patient activation assessed (PAM-13)
Dykes (2020)	Fall TIPS programme with automated CDS linking risk factors to interventions; individualised plans reviewed with patients and families each shift
Hill (2015)	Safe Recovery programme: Individualised education (video + face-to-face) delivered within 48 h by PT/OT; explanation of personal risk and development of an action plan with patients
Morris (2024)	Allied health assistant–delivered education programme: Structured scripted conversation and educational materials; reinforcement of personal fall risk awareness integrated into usual care; simple goal-oriented messages provided
Guo (2023)	Patient engagement framework–based programme: Tablet-based education and patient self-assessment; nurse–patient co-analysis; automated individualised plans with behavioural rehearsal and peer engagement
Radecki (2020)	PFAT: Patient-completed self-assessment followed by nurse–patient discussion and co-production of a personalised bedside plan

CDS, clinical decision support; EHR, electronic health record; PFAT, Patient Fall Assessment Tool; Fall TIPS, Tailoring Interventions for Patient Safety; PAM-13, 13-item Patient Activation Measure.

### 3.2 Patient engagement across the four phases

#### 3.2.1 Overall structure

Patient engagement varied across the four phases of fall prevention process (risk awareness, co-planning, goal setting, and self-management), with substantial differences in the depth and structure of engagement across interventions (Table 3). Engagement levels ranged from Level 1 (passive engagement) to Level 2 (shared decision-making) and Level 3 (autonomous decision-making). However, higher levels of engagement were observed only in a limited number of phases and interventions. Analysis of verbatim descriptions of intervention content (Supplementary Table S2) demonstrated considerable variation in how patient engagement was described and operationalised across studies.

**Table 3:** Patient Engagement Levels Across Fall Prevention Phases.

Author	Risk awareness				Co-planning				Goal setting				Self-management			Overall
	Assessment tool/method	Assessor	Initial assessment	Reassessment	EL*	Plan developer	Plan modification	Modification details	EL	Goal setting	Goal setter	Goal type	EL	Action	EL	Overall EL*
Dykes (2017)	Morse Fall Scale	Nurse	At admission (≤24 h)	Not described	L1	Nurse	Yes	At bedside	L1	Not described	Not specified (plan reviewed jointly)	Behaviour-oriented	L1	No	L1	L1
Duckworth (2019)	Morse Fall Scale + CDS	Nurse	At admission (≤24 h)	Not described (adherence monitoring reported)	L1	Nurse	Yes	Nurse-led updates	L1	Implicit (inferred from adherence process)	Not specified	Behaviour-oriented (implicit)	L1	No	L1	L1
Christiansen (2020)	Morse Fall Scale (via Fall TIPS)	Nurse	At admission (≤24 h)	Yes	L2	Nurse	Implied update of risk & plan	Updated risk & plan	L2	Not described	Not specified	Behaviour-oriented	L2	Partial	L3	L3
Dykes (2020)	Morse Fall Scale + CDS (Fall TIPS)	Nurse	At admission (≤24 h)	Yes (each shift)	L1	Nurse	Yes (each shift)	Shift-based updates	L2	Explicit	Joint (patient + healthcare professionals + family)	Behaviour-oriented	L2	No	L2	L2
Hill (2015)	Ward standard assessment	Rehabilitation staff (PT/OT)	Early hospitalisation (≤48 h)	Not specified	L2	Rehabilitation staff (PT/OT)	Yes (session-based)	Session-based	L2	Explicit (education-driven)	Joint (patient + healthcare professionals)	Mixed (behaviour- and outcome-oriented)	L3	Partial	L3	L3
Morris (2024)	Ward standard assessment (not intervention-specific)	Nurse / usual care staff	Early hospitalisation (≤48 h)	Not specified	L1	Nurse / usual care staff	Not specified	Not described	L2	Implicit (education-driven; simple goals provided)	Healthcare professionals	Education-focused (knowledge and awareness enhancement)	L2	Partial	L2	L2
Guo (2023)	Electronic self-assessment + nurse review	Patient + Nurse	Within 4 h of admission	Yes	L3	Patient + Nurse	Yes	Daily adjustment	L3	Implicit	Joint (patient + nurse)	Behaviour-oriented	L3	Yes	L3	L3
Radecki (2020)	JHFRAT + PFAT self-assessment	Patient + Nurse	Within 24h	Yes	L2	Patient + Nurse	Yes (board updated)	Board updated	L2	Not described	Not specified	Behaviour-oriented	L2	Partial	L2	L2

CDS, clinical decision support; EHR, electronic health record; JHFRAT, Johns Hopkins Fall Risk Assessment Tool; PFAT, Patient Fall Assessment Tool; PT, Physical Therapist; OT, Occupational Therapist.

* EL, Engagement Level, L1, passive engagement (clinician-led decision-making); L2, shared decision-making (patients participate in decision-making with healthcare professionals); L3, autonomous decision-making (patients take a leading role in decision-making and behavioural adjustment).

* Overall EL, Overall Engagement Level, indicates the highest engagement level achieved across the four phases.

#### 3.2.2 Risk awareness and risk assessment

All included studies incorporated a risk awareness or risk assessment component; however, in most interventions this process was initiated and led by healthcare professionals, corresponding to Level 1 (passive engagement). Patients were primarily involved in receiving information about their fall risk and confirming their understanding, with little opportunity to influence the assessment process. Verbatim descriptions frequently emphasised risk communication and confirmation of understanding rather than active participation (Supplementary Table S2).

Higher levels of engagement were observed in several studies through different approaches. Within the Fall TIPS framework, (Christiansen et al., 2020) involved patients in reviewing and interpreting their individualised risk profiles, corresponding to Level 2 (shared decision-making). Similarly, the Safe Recovery programme (Hill et al., 2015) incorporated dialogue between rehabilitation staff and patients regarding individual fall risk, although the assessment process remained clinician-directed. The highest level of engagement during this phase was observed in the studies by (Guo et al., 2023) and (Radecki et al., 2020), which incorporated patient self-assessment as part of risk identification. Patients actively identified personal risk factors and discussed these with healthcare professionals, corresponding to Level 2 (shared decision-making) to Level 3 (autonomous decision-making). Verbatim descriptions such as “patients independently assess” and “patient-directed assessment” reflected this increased patient agency (Supplementary Table S2).

#### 3.2.3 Co-planning

All included studies developed individualised fall prevention plans; however, the extent of patient involvement in planning varied considerably across interventions. In most Fall TIPS-based programmes, prevention plans were developed by healthcare professionals and subsequently shared with patients, corresponding to Level 1 (passive engagement). Patients primarily participated by reviewing or agreeing with the proposed plans rather than contributing to their development. Verbatim descriptions such as “nurses complete the plan” reflected this clinician-led approach (Supplementary Table S2).

Greater patient involvement was observed in several interventions. Within the Fall TIPS framework, (Christiansen et al., 2020) and (Dykes et al., 2020) actively involved patients in reviewing and discussing individualised prevention plans, corresponding to Level 2 (shared decision-making). Similarly, (Hill et al., 2015) and (Guo et al., 2023) described collaborative planning processes in which patients and healthcare professionals jointly interpreted fall risk and co-developed preventive strategies. Expressions such as “jointly developed” and “developed together” reflected this collaborative approach (Supplementary Table S2).

(Morris et al., 2024) incorporated structured educational discussions and simple goal-oriented planning; however, patients had limited influence over the planning process, corresponding to Level 2 (shared decision-making). Likewise, (Radecki et al., 2020) incorporated patient self-assessment and discussion with clinicians, corresponding to Level 2 (shared decision-making).

#### 3.2.4 Goal setting

Goal setting represented the greatest structural gap across the included interventions. Only a small number of studies explicitly described goal-setting processes (Hill et al., 2015; Dykes et al., 2020; Guo et al., 2023; Morris et al., 2024), whereas most interventions incorporated goals only implicitly within education or care plans. Consequently, patient engagement during this phase remained predominantly between Level 1 (passive engagement) and Level 2 (shared decision-making).

Higher levels of engagement were observed in the studies by (Hill et al., 2015) and (Guo et al., 2023), in which patients actively participated in setting behavioural goals and developing action plans, corresponding to Level 3 (autonomous decision-making). Verbatim descriptions such as “patients set behavioural goals”, “written action plan”, and “patient-selected actions” reflected active patient involvement in directing subsequent preventive behaviours (Supplementary Table S2).

By contrast, (Morris et al., 2024) primarily presented goals through healthcare professionals with limited patient involvement, corresponding to Level 1 (passive engagement) to Level 2 (shared decision-making). Although (Christiansen et al., 2020) demonstrated relatively high engagement during earlier phases, explicit goal-setting processes were not described, indicating that higher engagement in risk awareness or co-planning did not necessarily extend to goal setting.

#### 3.2.5 Self-management

Self-management was less consistently implemented than the earlier phases. Nevertheless, all included studies reported some degree of patient engagement in fall prevention behaviours. In most interventions, however, patients primarily followed predefined prevention plans rather than actively monitoring and adapting their own behaviours. Patient engagement during this phase was predominantly between Level 1 (passive engagement) and Level 2 (shared decision-making). Verbatim descriptions such as “plan adherence” and “following the plan” reflected passive execution rather than active self-regulation (Supplementary Table S2). (Hill et al., 2015) encouraged patients to implement self-directed behaviours based on individualised action plans, corresponding to Level 3 (autonomous decision-making) engagement, although ongoing self-monitoring and iterative adjustment were not fully structured. (Christiansen et al., 2020) also demonstrated higher engagement, inferred from improvements in patient activation, suggesting greater behavioural involvement despite limited descriptions of explicit self-management processes. The most comprehensive approach was reported by (Guo et al., 2023), which incorporated ongoing self-assessment, daily behavioural modification, and continuous evaluation, representing Level 3 (autonomous decision-making). Verbatim descriptions such as “patients participated in implementation and dynamic evaluation” reflected active behavioural regulation (Supplementary Table S2). By contrast, although (Radecki et al., 2020) incorporated patient self-assessment and participation in implementation, decision-making remained shared with healthcare professionals, and fully autonomous self-management was not achieved.

### 3.3 Cognitive eligibility and family involvement

Patient engagement was strongly dependent on cognitive function (Supplementary Table S3). In several studies (Hill et al., 2015; Radecki et al., 2020; Guo et al., 2023; Morris et al., 2024), cognitive status was explicitly defined as an eligibility criterion, limiting engagement to cognitively intact patients. Although these designs enable deeper forms of engagement, they also introduce inherent structural constraints by selectively including patients with sufficient cognitive capacity. By contrast, (Dykes et al., 2017; 2020) and (Duckworth et al., 2019) imposed no explicit cognitive restrictions; however, family involvement was incorporated, either implicitly or explicitly, as a compensatory mechanism for patients with cognitive impairment.

Inconsistencies were also observed between eligibility criteria for intervention participation and the cognitive requirements for outcome measurement. For example, (Christiansen et al., 2020) applied no explicit restrictions on participation, whereas patient-reported outcomes were limited to cognitively able participants. This discrepancy suggests that ‘eligibility for participation’ and ‘eligibility for evaluation’ were operationalised using different criteria, potentially creating a gap between actual engagement and measured outcomes.

The role of family members varied widely across studies, ranging from passive recipients of information (Dykes et al., 2017), to proxy participants (Duckworth et al., 2019), and active partners in the care process (Dykes et al., 2020). However, no consistent definition of family involvement was identified. Family roles ranged from information exchange to participation in decision-making, with limited consistency across studies (Supplementary Table S3). In particular, proxy engagement was not clearly operationalised, and the extent of family involvement in decision-making varied substantially. Furthermore, even in studies that excluded patients with cognitive impairment, family involvement was not consistently incorporated as a structured alternative to support engagement.

### 3.4 Information-sharing modalities and action linkage

All studies incorporated information-sharing strategies; however, the extent to which information or care plans were explicitly connected to patient actions (action linkage) varied considerably across interventions (Table 4).

**Table 4:** Information-Sharing Media, Formats, and Implementation Characteristics in Patient-Engaged Fall-Prevention Programmes.

Author (Year)	Media type	Format	Display location	Family access	Interactivity	Goal setting	Action linkage	Updating / feedback	Timing of delivery
Dykes (2017)	Laminated poster	Structured (risk + tailored plan)	Bedside	Limited (implicit)	Moderate	Not described	Indirect	Not specified	At admission
Duckworth (2019)	EHR / poster / bedside display	Structured (CDS-linked)	Bedside	Limited	Moderate	Implicit	Indirect	Adherence monitoring	At admission
Christiansen (2020)	EHR-integrated display	Structured (CDS + activation focus)	Bedside	Limited	Moderate	Not described	Limited	Not specified	At admission
Dykes (2020)	Poster / EHR / bedside display	Structured (CDS-linked, individualised)	Bedside	Yes (explicit)	High	Explicit	Strong	Shift-based updating	Admission + ongoing
Hill (2015)	Video (DVD) + workbook	Tailored, behaviour change–based	Bedside (non-fixed)	Limited	Very high	Explicit (behavioural goals)	Strong	Session-based reinforcement	Within 48h
Morris (2024)	Scripted conversation + pamphlet	Structured (motivational interviewing)	Bedside (non-fixed)	Limited	Very high	Explicit (goal-oriented plan with behavioural cues)	Moderate	Follow-up (e.g., day 5)	Within 48h
Guo (2023)	Tablet + self-assessment tool + bedside cards	Structured (engagement framework–based)	Bedside + digital	Not specified	Very high	Explicit (patient-selected actions)	Very strong	Daily adjustment / feedback	Within 4h
Radecki (2020)	Paper-based PFAT + laminated board	Structured (self-assessment + co-production)	Bedside	Not specified	Moderate	Implicit	Limited	Plan updated as needed	Within 24h

CDS, clinical decision support; EHR, electronic health record; DVD, Digital Versatile Disc; PFAT, Patient Fall Assessment Tool.

Interactivity refers to the degree of bidirectional communication and patient involvement in decision-making processes.

Goal setting indicates whether explicit patient-centred behavioural goals were described.

Updating/feedback denotes whether and how intervention content was revised during hospitalisation.

Action linkage refers to the extent to which provided information or plans are directly connected to specific patient behaviours or behavioural execution.

In several studies, information was delivered through bedside displays, such as laminated posters or Electronic Health Record (EHR)-linked interfaces (Dykes et al., 2017; Duckworth et al., 2019; Christiansen et al., 2020; Dykes et al., 2020). These interventions provided structured information on fall risk and prevention plans; however, goal setting was often absent or implicit, and the link to patient action remained indirect. Although (Christiansen et al., 2020) incorporated an activation-focused approach, a clear connection between information and patient behaviour was not established. Updating and feedback mechanisms were frequently unspecified, indicating predominantly unidirectional information delivery.

By contrast, behaviour-change-oriented interventions, particularly those by (Hill et al., 2015) and (Morris et al., 2024), explicitly integrated goal setting and action planning. In (Hill et al., 2015), patients actively participated in defining behavioural goals, whereas in (Morris et al., 2024), goal-oriented plans were largely structured and guided by healthcare professionals, with limited patient involvement in shaping actions.

(Guo et al., 2023) demonstrated a more integrated model in which digital tools and self-assessment enabled patients to select actions and adjust behaviours over time. Goal setting was explicit and patient-directed, with strong action linkage and daily feedback, indicating close alignment between information and behavioural regulation.

In (Radecki et al., 2020), patient self-assessment supported understanding and participation; however, goal setting remained implicit, and both action linkage and feedback were limited.

The Fall TIPS intervention (Dykes et al., 2020) represented an intermediate model that combined structured information delivery with explicit goal setting, strong action linkage, and continuous updating through shift-based review.

Overall, although information sharing was universal, interventions differed substantially in the extent to which information was translated into patient action through explicit goal setting, action planning, and ongoing feedback. These findings highlight that the effectiveness of information sharing depends not only on the provision of information but also on how explicitly it supports behavioural implementation.

## 4 Discussion

This scoping review synthesised evidence from eight studies on patient-engaged fall prevention in hospital settings. The findings indicate that patient engagement is unevenly distributed across interventions, varies in depth across phases of the prevention process, and was shaped by structural constraints. Although information sharing was incorporated into all interventions, its contribution to patient engagement depended on whether it was explicitly linked to goal setting, action selection, and feedback. These findings suggest that patient engagement extends beyond the provision of information and should be understood as a structured process that supports progression from information sharing to action. Furthermore, engagement was described using diverse and often ambiguous terminology, highlighting the need for an operational framework (Carman et al., 2013; Castro et al., 2016). Accordingly, this review reconceptualises patient engagement as a phase-dependent process that is shaped by the structural organisation of interventions.

### 4.1 Phase-dependent distribution of engagement

The most important finding of this review is that patient engagement was unevenly distributed across the phases of inpatient fall prevention. Engagement was most evident during risk awareness and co-planning, whereas explicit goal setting and self-management were less frequently incorporated. These findings suggest that patient engagement is not a static characteristic of an intervention but varies according to where patients are involved in the prevention process.

This pattern is consistent with literature describing patient engagement as a dynamic and evolving process involving collaborative relationships, active participation, and goal-oriented behaviours (Jiang et al., 2025; González et al., 2026; Graffigna et al., 2015). Rather than examining engagement as a single overall construct, the present review demonstrates that the location of engagement within the prevention pathway is also important. Most interventions supported participation during assessment and planning, whereas relatively few extended participation into patient-led goal setting or self-management. Thus, the findings highlight potential gaps in the later phases through which information and shared planning might otherwise be translated into preventive action.

### 4.2 Engagement as a structurally constrained process

Another key implication of this review is that patient engagement is shaped not only by individual patient capacity or motivation but also by intervention design and healthcare system conditions (Carman et al., 2013; Ocloo and Matthews, 2016; Hickmann et al., 2022). Contemporary conceptualisations of patient engagement emphasise that engagement is a relational and context-dependent process that emerges through interactions among patients, healthcare professionals, and healthcare systems rather than solely from individual patient characteristics (Hickmann et al., 2022). In particular, the limited integration of goal setting and self-management suggests that restricted engagement in later phases reflects not a deficit on the part of patients, but the absence of system-level structures that support participation at these stages.

This interpretation is consistent with previous reviews indicating that patient engagement is enabled or constrained by organisational and institutional contexts (Ocloo and Matthews, 2016; Bombard et al., 2018). Engagement does not occur spontaneously but requires structures that actively facilitate participation. In acute care settings, time constraints and workload pressures often limit opportunities for sustained interactions with patients (Bolster and Manias, 2010; Tobiano et al., 2015), contributing to the concentration of engagement in the earlier phases. Rehabilitation settings may more readily incorporate goal-oriented and dialogic processes (Levack et al., 2016); however, not all interventions in these contexts achieved higher levels of engagement, suggesting that intervention design, rather than setting alone, is the more important determinant.

### 4.3 Information sharing as a necessary but insufficient condition

All included studies incorporated some form of information sharing; however, in most cases, this remained largely unidirectional and did not sufficiently translate into patient decision-making or behavioural change.

This finding is consistent with evidence on the implementation of patient-centred care in acute settings (Epstein and Street, 2011; Kitson et al., 2013). Although the principles of patient-centred care are widely endorsed, implementation is often constrained by organisational culture, education, resources, and workflow structures. Access to information is generally ensured in fall prevention, but it does not automatically lead to active involvement in decision-making or sustained behavioural adjustment (Yi et al., 2026).

Information sharing should therefore be regarded as a necessary but insufficient condition for patient engagement. Meaningful participation requires information-sharing to be integrated with interactive processes such as dialogue, decision support, and action planning (Elwyn et al., 2012; Stacey et al., 2017). As shown in Supplementary Table S2, engagement-related terminology was used inconsistently across studies; however, such expressions do not necessarily indicate actual involvement in decision-making or behavioural change, reflecting ongoing conceptual ambiguity.

Importantly, (Radecki et al., 2020) found that increased perceived involvement in a prevention plan did not significantly improve adherence to preventive behaviours, suggesting that awareness or perceived engagement alone may be insufficient to achieve behavioural change. Rather than focusing on the mode of information delivery, the critical issue is whether interventions enable patients to interpret information, make choices, and translate them into action. This interpretation is consistent with contemporary behavioural theories, which emphasise that information alone is insufficient to change behaviour (Yi et al., 2026; Takase, 2026). (Takase, 2026), using a revised Protection Motivation Theory, demonstrated that patients’ adherence depends not only on understanding fall-prevention information but also on self-efficacy, response efficacy, and supportive nurse–patient relationships. These findings reinforce the present review by suggesting that information sharing is most effective when it is explicitly linked to goal setting, action planning, and self-management.

### 4.4 Goal setting as the missing link between knowledge and action

Goal setting was the least clearly defined component across the included studies. Although many interventions were successful in providing information, the translation of this information into explicit behavioural goals was often insufficient.

This finding is consistent with behavioural change theory, which emphasises the role of goal setting in linking knowledge to action (Michie et al., 2011). Comparisons across studies, particularly between (Dykes et al., 2017; 2020) and (Guo et al., 2023), suggest that behavioural change depends not simply on the presence of patient engagement but on the extent to which engagement is integrated into the care process.

Therefore, the key issue in intervention design is not only what information is provided but also how it is translated into actionable goals.

### 4.5 Cognitive function and family involvement as determinants of the scope of engagement

Patient engagement was strongly influenced by cognitive function, although studies addressed this constraint in different ways. Some interventions excluded patients with cognitive impairment (Hill et al., 2015; Radecki et al., 2020; Guo et al., 2023; Morris et al., 2024), whereas others included these patients and relied on family members as proxy participants (Dykes et al., 2017; Duckworth et al., 2019; Christiansen et al., 2020; Dykes et al., 2020). In addition, Christiansen et al. (2020) imposed no restrictions on intervention participation but limited outcome measurements to cognitively able patients

These patterns indicate that for patients with cognitive impairment or communication difficulties, engagement is shaped not only by individual capacity but also by relational and structural factors, including proxy involvement. Consistent with literature, cognitive impairment represents a major constraint on participation, whereas family members may act as substitutes in decision-making processes (Elwyn et al., 2012; Carman et al., 2013). However, the roles of proxy participants and the distribution of decision-making responsibilities remain poorly defined.

Taken together, these findings suggest that for patients requiring proxy support, patient engagement should be understood as a relational process involving patients, families, and healthcare professionals rather than an individual attribute. Future interventions should therefore incorporate structured approaches to proxy and supported decision-making tailored to patients’ cognitive capacity.

### 4.6 Digital interventions: potential and limitations

Digital technologies can enhance patient engagement, but their impact is not guaranteed. In the digital interventions included in this review (Guo et al., 2023), digital tools facilitated access to information and enabled self-assessment and feedback, suggesting a more interactive structure than traditional unidirectional approaches.

However, digital technology alone did not ensure greater involvement in decision-making or behavioural change. Rather, its effectiveness depended on integration into clinical processes. This finding is consistent with evidence indicating that the impact of digital interventions is determined by their design and implementation rather than by the technology itself (Barello and Graffigna, 2015; Murray et al., 2016).

Digital interventions limited to information delivery tended to support only passive forms of engagement, whereas higher levels of engagement required the incorporation of decision support and behavioural adjustment functions. Moreover, their effectiveness may be influenced by patients’ cognitive capacity and digital literacy, potentially widening disparities in participation.

These findings suggest that digital technologies should not be viewed as standalone solutions but as components of integrated intervention processes that combine information sharing, decision support, goal setting, and feedback.

### 4.7 Implications for practice and research

These findings suggest several priorities for designing patient-engaged fall prevention interventions. First, engagement should be conceptualised and implemented as a phase-integrated process rather than as a single construct. Second, explicit goal-setting components are required to translate information into actionable behaviours. Third, inclusive models should support participation among patients with cognitive impairment through the systematic incorporation of family roles. Fourth, information-sharing strategies should be integrated with decision support and behavioural regulation rather than implemented as standalone components.

In addition, evaluation frameworks should move beyond measuring the degree of engagement to examine how it is distributed across phases of care. The phase-based framework proposed in this review offers a practical approach to capturing the structure and depth of engagement in complex clinical interventions. This perspective aligns with previous work emphasising the organisational and contextual nature of patient engagement (Carman et al., 2013; Ocloo and Matthews, 2016).

Overall, this review highlights the need to reconceptualise patient engagement as a phase-dependent and context-sensitive process rather than a uniformly applied ideal. This approach provides a useful basis for the design and evaluation of interventions.

## 5 Limitations

This study had several limitations. First, as a scoping review, it did not assess the methodological quality of the included studies or synthesise effect sizes. Therefore, causal relationships between levels of patient engagement and clinical outcomes could not be established.

Second, the classification of patient engagement was based on an operational framework derived from study descriptions and therefore involved interpretive judgement. In many original studies, ‘engagement’ was not explicitly defined. Although classifications were informed by verbatim descriptions (Supplementary Table S2), variability in terminology and reporting may have affected consistency.

Third, the included studies were predominantly conducted in acute care and rehabilitation settings and were geographically concentrated, which may limit generalisability to other healthcare systems and cultural contexts.

Fourth, assessments of engagement relied primarily on the intervention design and reported processes rather than direct measures of patients’ experiences. In studies with limited patient-reported outcomes, the level of engagement may have been either underestimated or overestimated.

Fifth, analyses of cognitive function and family involvement (Supplementary Table S3) were constrained by heterogeneity in reporting across studies. Interventions involving patients with cognitive impairment were limited, and family roles were inconsistently defined, restricting comparability. In addition, this review included only English-language publications and may be subject to language bias.

Finally, because all included studies evaluated structured interventions, the observed levels of patient engagement reflected intervention design rather than patients’ usual participation in routine clinical care. Consequently, the findings should not be interpreted as representing everyday patient involvement outside the evaluated interventions.

## 6 Conclusions

This scoping review demonstrates that patient engagement in inpatient fall prevention is best understood as a phase-dependent process rather than a single, uniform construct. Although engagement was consistently incorporated during risk awareness and co-planning, goal setting and self-management remained underdeveloped, highlighting the need to move beyond information sharing towards action-oriented support. Future interventions should adopt phase-integrated approaches that explicitly incorporate goal setting and accommodate patients' varying opportunities and capacities for participation. The proposed framework provides a practical basis for designing and evaluating patient engagement interventions in inpatient fall prevention.

## Ethical approval

Ethical approval was not required for this study because it was based on a review of published literature and did not involve human participants.

## Funding

This work was supported by the Japan Society for the Promotion of Science KAKENHI, Grant-in-Aid for Scientific Research (B) (22H03377).

## CRediT authorship contribution statement

**Sayaka Nishihara:** Writing – review & editing, Writing – original draft, Visualization, Methodology, Investigation, Formal analysis, Data curation, Conceptualization. **Masae Satoh:** Writing – review & editing, Writing – original draft, Visualization, Supervision, Project administration, Methodology, Funding acquisition, Formal analysis, Data curation, Conceptualization. **Yuriko Iijima:** Writing – review & editing. **Mika Katoh:** Writing – review & editing, Investigation. **Hiromi Shimmura:** Writing – review & editing. **Rika Kawahara:** Writing – review & editing, Investigation. **Natsuko Shimazawa:** Writing – review & editing, Investigation.

## Declaration of competing interest

The authors declare that they have no known competing financial interests or personal relationships that could have appeared to influence the work reported in this paper.

## Data Availability

Data sharing is not applicable to this article because no datasets were generated during the current study.

## References

[bib0001] Alvarado N., Lynn McVey L., Healey F., Dawn Dowding D., Zaman H., Cheong V.H., Gardner P., Lynch A., Nick Hardiker N., Rebecca Randell R. (2025). Implementing recommended falls prevention practices for older patients. BMJ Open..

[bib0002] Bandura A. (1997). Self-Efficacy: The Exercise of Control.

[bib0003] Barello S., Graffigna G. (2015). Patient engagement in healthcare: pathways for effective medical decision making. Neuropsychol. Trends.

[bib0004] Bernet N.S., Everink I.H., Schols J.M., Halfens R.J., Richter D., Hahn S. (2022). Hospital performance comparison of inpatient fall rates: the impact of risk adjusting for patient-related factors: a multicentre cross-sectional survey. BMC Health Serv. Res..

[bib0005] Bolster D., Manias E. (2010). Person-centred interactions between nurses and patients during medication activities in an acute hospital setting: qualitative observation and interview study. Int. J. Nurs. Stud..

[bib0006] Bombard Y., Baker G.R., Orlando E., Fancott C., Bhatia P., Casalino S., Onate K., Denis J.L., Pomey M.P. (2018). Engaging patients to improve quality of care: a systematic review. Implement. Sci..

[bib0007] Carman K.L., Dardess P., Maurer M., Sofaer S., Adams K., Bechtel C., Sweeney J. (2013). Patient and family engagement: a framework for understanding the elements and developing interventions and policies. Health Aff..

[bib0008] Castro E.M., Van Regenmortel T., Vanhaecht K., Sermeus W., Van Hecke A. (2016). Patient empowerment, patient participation and patient-centeredness in hospital care: a concept analysis based on a literature review. Patient. Educ. Couns..

[bib0009] Charles C., Gafni A., Whelan T. (1997). Shared decision-making in the medical encounter: what does it mean? (or it takes at least two to tango). Soc. Sci. Med..

[bib0010] Christiansen T., Lipsitz S., Scanlan M., Yu S.P., Lindros M.E., Leung W.Y., Adelman J., Bates D.W., Dykes P.C. (2020). Patient activation related to fall prevention: a multisite study. Jt. Comm. J. Qual. Patient. Saf..

[bib0011] Duckworth M., Adelman J., Belategui K., Feliciano Z., Jackson E., Khasnabish S., Lehman I.-F.S., Lindros M.E., Mortimer H., Ryan K., Scanlan M., Berger Spivack L., Yu S.P., Bates D.W., Dykes P.C. (2019). Assessing the effectiveness of engaging patients and their families in the three-step fall prevention process across modalities of an evidence-based fall prevention toolkit: an implementation science study. JMIR Med. Inform..

[bib0012] Dykes P.C., Duckworth M., Cunningham S., Dubois S., Driscoll M., Feliciano Z., Ferrazzi M., Fevrin F.E., Lyons S., Lindros M.E., Monahan A., Paley M.M., Jean-Pierre S., Scanlan M. (2017). Pilot testing fall TIPS (Tailoring Interventions for Patient Safety): a patient-centered fall prevention toolkit. Jt. Comm. J. Qual. Patient. Saf..

[bib0013] Dykes P.C., Adelman J.S., Alfieri L., Bogaisky M., Carroll D., Carter E., Duckworth M., Ives Erickson J.R., Flaherty L.M., Hurley A.C., Jackson E., Khasnabish S., Lindros M.E., Manzano W., Scanlan M., Spivack L.B. (2019). The fall TIPS (Tailoring Interventions for Patient Safety) program: a collaboration to end the persistent problem of patient falls. Nurse Lead..

[bib0014] Dykes P.C., Burns Z., Adelman J., Benneyan J., Bogaisky M., Carter E., Ergai A., Lindros M.E., Lipsitz S.R., Scanlan M., Shaykevich S., Bates D.W. (2020). Evaluation of a patient-centered fall-prevention tool kit to reduce falls and injuries: a nonrandomized controlled trial. JAMA Netw. Open..

[bib0015] Dykes P.C., Mica Curtin-Bowen M., Lipsitz S., Franz C., Adelman J., Adkison L., Bogaisky M., Carroll D., Carter E., Herlihy L., Lindros M.E., Ryan V., Scanlan M., Walsh M.-A., Wien M., Bates D.W. (2023). Cost of inpatient falls and Cost-benefit analysis of implementation of an evidence-based fall prevention program. JAMA Health Forum..

[bib0016] Elwyn G., Frosch D., Thomson R., Joseph-Williams N., Lloyd A., Kinnersley P., Cording E., Tomson D., Dodd C., Rollnick S., Edwards A., Barry M. (2012). Shared decision making: a model for clinical practice. J. Gen. Intern. Med..

[bib0017] Epstein R.M., Street R.L. (2011). The values and value of patient-centered care. Ann. Fam. Med..

[bib0018] González M.L.S., Nitsch K., Carnahan N., Aaron R.V., Hosey M.M., Schechter N. (2026). Patient engagement in inpatient rehabilitation: a scoping review of measures and evolving conceptualizations. Rehabil. Psychol..

[bib0019] Graffigna G., Barello S., Bonanomi A., Lozza E. (2015). Measuring patient engagement: development and psychometric properties of the patient health engagement (PHE) Scale. Front. Psychol..

[bib0020] Greene J., Hibbard J.H. (2012). Why does patient activation matter? An examination of the relationships between patient activation and health-related outcomes. J. Gen. Intern. Med..

[bib0021] Guo X., Wang Y., Wang L., Yang X., Yang W., Lu Z., He M. (2023). Effect of a fall prevention strategy for the older patients: a quasi-experimental study. Nurs. Open..

[bib0022] Hibbard J.H., Stockard J., Mahoney E.R., Tusler M. (2004). Development of the patient Activation Measure (PAM): conceptualizing and measuring activation in patients and consumers. Health Serv. Res..

[bib0023] Hickmann E., Richter P., Schlieter H. (2022). All together now - patient engagement, patient empowerment, and associated terms in personal healthcare. BMC Health Serv. Res..

[bib0024] Hill A.M., McPhail S.M., Waldron N., Etherton-Beer C., Ingram K., Flicker L., Bulsara M., Haines T.P. (2015). Fall rates in hospital rehabilitation units after individualised patient and staff education programmes: a pragmatic, stepped-wedge, cluster-randomised controlled trial. Lancet.

[bib0025] Hirose M., Nakabayashi N., Fukuda S., Yamaguchi S., Igawa M., Egami K., Honda J., Shima H. (2018). Additional medical costs due to hospital-acquired falls. J. Patient. Saf..

[bib0026] Isaac J.P., Gormley K.J., Goswami N., AbuRuz M., Abdulnaby E., Rothwell A., Carandang M.T., Hamdan A. (2026). Perspectives on fall prevention among older patients and healthcare providers. Clin. Interv. Aging.

[bib0027] Japan Council for Quality Health Care (2025). Project to Collect Medical Near-Miss/Adverse Event Information 2024 Annual Report.

[bib0028] Jiang H., Lin B., Liu Z. (2025). Patient engagement in rehabilitation: an evolutionary concept analysis. Clin. Rehabil..

[bib0029] Kitson A., Marshall A., Bassett K., Zeitz K. (2013). What are the core elements of patient-centred care? A narrative review and synthesis of the literature from health policy, medicine and nursing. J. Adv. Nurs..

[bib0030] Levack W.M., Weatherall M., Hay-Smith J.C., Dean S.G., McPherson K., Siegert R.J. (2016). Goal setting and strategies to enhance goal pursuit in adult rehabilitation: summary of a Cochrane systematic review and meta-analysis. Eur. J. Phys. Rehabil. Med..

[bib0031] Li L.W., Wong V.W.S., Li H.Y.M., Cheung C.C.Y., Yeung K.M. (2025). Fall prevention programme for postoperative older adults at a private hospital. Asian J. Gerontol. Geriatr..

[bib0033] McKercher J.P., Peiris C.L., Hill A.M., Peterson S., Thwaites C., Fowler-Davis S., Morris M.E. (2024). Hospital falls clinical practice guidelines: a global analysis and systematic review. Age Ageing.

[bib0034] Michie S., Van Stralen M.M., West R. (2011). The behaviour change wheel: a new method for characterising and designing behaviour change interventions. Implement. Sci..

[bib0035] Morris M.E., Thwaites C., Lui R., McPhail S.M., Haines T., Kiegaldie D., Heng H., Shaw L., Hammond S., McKercher J.P., Knight M., Carey L.M., Gray R., Shorr R., Hill A.-M. (2024). Preventing hospital falls: feasibility of care workforce redesign to optimise patient falls education. Age Ageing.

[bib0036] Morris M.E., Said C.M., Haines T., Heng H.W.F., Batchelor F., Hutchinson A.M., McKercher J.P., Semciw A.I., Hill A.M., Peterson S., Kane R., Fowler-Davis S., Campbell S., Sherrington C., Gilmartin-Thomas J., Phan U., Hospital Falls Reference Standard Consensus Group (2025). Reference standard for the prevention and management of hospital falls: a multidisciplinary Delphi consensus study. BMJ Open..

[bib0037] Mou Q., You M., Tao L., Li J., Jiang Y., Jiang X. (2026). Attitudes and participation in fall risk management. PLoS. One.

[bib0038] Munn Z., Peters M.D.J., Stern C., Tufanaru C., McArthur A., Aromataris E. (2018). Systematic review or scoping review? Guidance for authors when choosing between a systematic or scoping review approach. BMC Med. Res. Methodol..

[bib0039] Murray E., Hekler E.B., Andersson G., Collins L.M., Doherty A., Hollis C., Rivera D.E., West R., Wyatt J.C. (2016). Evaluating digital health interventions: key questions and approaches. Am. J. Prev. Med..

[bib0040] Ocloo J., Matthews R. (2016). From tokenism to empowerment: progressing patient and public involvement in healthcare improvement. BMJ Qual. Saf..

[bib0041] Pollock D., Peters M., Tricco A., Munn Z., Jia R., Alexander L., Pieper D., Evans C., Godfrey C., Brandão de Moraes E., Saran A., Campbell F., Khalil H., Aromataris E., Lockwood C., Porritt K., Pilla B., Jordan Z. (2024). JBI Manual for Evidence Synthesis.

[bib0042] Popay J., Roberts H., Sowden A., Petticrew M., Arai L., Rodgers M., Britten N., Roen K., Duffy S. (2006). Guidance on the Conduct of Narrative Synthesis in Systematic Reviews.

[bib0043] Radecki B., Keen A., Miller J., McClure J.K., Kara A. (2020). Innovating fall safety: engaging patients as experts. J. Nurs. Care Qual..

[bib0044] Satoh M., Nakahori T., Shimada T. (2026). Risk-adjusted inpatient falls as indicators of health system performance during the COVID-19 pandemic. Healthcare.

[bib0045] Stacey D., Légaré F., Lewis K., Barry M.J., Bennett C.L., Eden K.B., Holmes-Rovner M., Llewellyn-Thomas H., Lyddiatt A., Thomson R., Trevena L. (2017). Decision aids for people facing health treatment or screening decisions. Cochrane Database Syst. Rev..

[bib0046] Steinmetz C., Stenzel C., Sylvester M., Glage D., Linke A., Sadlonova M., Arnim C.A.F.A., Schnieder M., Valentová M., Heinemann S. (2025). Use of a technology-based fall prevention program with visual feedback. JMIR. Form. Res..

[bib0047] Sultana M., Taylor C., McPhee D., Chandler A., Hyde H., Yekinni I., Sayeed N., Bessey M. (2025). Reducing falls in inpatient older adults. J. Res. Nurs..

[bib0048] Takase M. (2023). Falls as the result of interplay between nurses, patient and the environment: using text-mining to uncover how and why falls happen. Int. J. Nurs. Sci..

[bib0049] Takase M. (2026). Decoding why patients follow-or do not follow-fall-prevention instructions. Int. J. Nurs. Stud..

[bib0050] Tan Y.F.C., Lai W.N., Soh S.L.H., Ho J., Zhao R.H., Low L.L. (2025). From research to practice: a pilot implementation study of a falls self-efficacy. Front. Health Serv..

[bib0051] Tobiano G., Marshall A., Bucknall T., Chaboyer W. (2015). Patient participation in nursing care on medical wards: an integrative review. Int. J. Nurs. Stud..

[bib0052] Tricco A.C., Lillie E., Zarin W., O’Brien K.K., Colquhoun H., Levac D., Moher D., Peters M.D.J., Horsley T., Weeks L., Hempel S., Akl E.A., Chang C., McGowan J., Stewart L., Hartling L., Aldcroft A., Wilson M.G., Garritty C., Lewin S., Godfrey C.M., Macdonald M.T., Langlois E.V., Soares-Weiser K., Moriarty J., Clifford T., Tunçalp Ö., Straus S.E. (2018). PRISMA extension for scoping reviews (PRISMA-ScR): checklist and explanation. Ann. Intern. Med..

[bib0053] Yasan C., Pretto G., Burton P. (2025). A high fall risk patient perspective. Nurs. Open..

[bib0054] Yi Y., Wen Y., Monteiro O., Wu X., Lam L.T. (2026). Fall prevention education among older hospital inpatients: a systematic review. Front Public Health.

[bib0055] Zhao Q., Zhou L., Guo J., Yang Y., Hou S. (2026). Beyond patient education. Front Public Health.

