# Supplementary material for: From information to action― a phase-based analysis of patient engagement in inpatient fall prevention: A scoping review
Source: Int J Nurs Stud Adv. 2026 Sep 10;11:100677. doi: 10.1016/j.ijnsa.2026.100677 (PMC13594758; doi:10.1016/j.ijnsa.2026.100677)
Supplement: Supplementary file 1 [file mmc1.docx]

**Supplementary Table S1 Search Strategy**

|  | **Search strategy** |
| --- | --- |
| **PubMed** | (((accidental falls [MeSH Terms]) OR (fall*[Title/Abstract])) AND ((Accident Prevention [MeSH Terms]) OR (prevention*[Title/Abstract]))) AND (((Patient Participation [MeSH Terms]) OR ((patient [Title/Abstract]) AND (participation [Title/Abstract] OR involvement [Title/Abstract] OR engagement [Title/Abstract] OR activation [Title/Abstract] OR empowerment [Title/Abstract]))) OR ("Patient and Public Involvement"[Title/Abstract])) |
| **Web of Science** | (((TI= (fall*)) OR AB= (fall*)) AND ((TI= (prevention*)) OR AB= (prevention*))) AND ((((TI= (patient)) OR AB= (patient)) AND ((TI= (participation OR involvement OR engagement OR activation OR empowerment)) OR (AB= (participation OR involvement OR engagement OR activation OR empowerment)))) OR ((TI= ("Patient and Public Involvement")) OR AB= ("Patient and Public Involvement"))) |
| **CINAHL** | (((MH "Accidental Falls" OR TI fall* OR AB fall*) AND (MH "Fall Prevention (Iowa NIC)" OR TI prevention* OR AB prevention*))) AND (((MH "Patient Participation+") OR (((TI patient) AND (TI (participation OR involvement OR engagement OR activation OR empowerment)))) OR (((AB patient) AND (AB (participation OR involvement OR engagement OR activation OR empowerment)))) OR ((TI ("Patient and Public Involvement")) OR (AB ("Patient and Public Involvement"))))) |

**Supplementary Table S2 Operational Classification of Patient Engagement Levels with Supporting Quotes**

| **Author**  **(Year)** | **Risk awareness** | **Co-planning** | **Goal setting** | **Self-management** | **Overall** |
| --- | --- | --- | --- | --- | --- |
| Dykes  (2017) | L1 | L1 | L1 | L1 | L1 |
|  | "3-step fall prevention process directly linked to nursing fall risk assessment work flow with critical engagement of the patient in the development of a personalized fall prevention plan" (Sidebar 1) | “Personalized bedside posters are generated based on an individual patient’s fall risk profile” (p.404) | " clinical decision support to link areas of risk with the interventions most likely to prevent a fall based on an individual patient's fall risk profile" (Figure 1 legends) | “Patients are more confident that they could identify their fall risk factors and things they should do” (p.409) “Improved knowledge of fall risk prevention plan and actions to reduce falls” (p.407) |  |
| Duckworth  (2019) | L1 | L1 | L1 | L1 | L1 |
|  | “nurses complete the Fall TIPS risk assessment and tailored plan” (p.3)  “linking the fall risk assessment to tailored interventions” (p.2) | “developing a tailored fall prevention plan” (p.2) “nurses complete the … tailored plan and document it in the EHR” (p.3) | “patients must be engaged throughout all 3 steps” (p.2) “patient/family knowledge of their personalized fall risk factors and prevention plan” (p.2) | “Does the patient/family member know their fall prevention plan?” (p.3) “implementing that plan consistently” (p.1) “patients did not follow their fall prevention plan” (p.2) |  |
| Christiansen  (2020) | L2 | L2 | L2 | L3 | L3 |
|  | “the nurse completes the fall risk assessment at the bedside with the patient” (p.129)  “engages patients in every step of the fall prevention process” (p.129) | “developing a personalized fall prevention plan” (p.129)  “the fall prevention plan is tailored to patient-specific risk factors and shared with the care team” (p.129) | “I am confident that I can take action that will help prevent or minimize some of the risk factors” (p.131) “Taking an active role… is the most important factor” (p.131) | “I am the person responsible for preventing myself from falling” (p.131)  “I know how to prevent myself from falling” (p.131) “I am confident that I can follow my fall prevention plan even during times of stress” （p.131）  “I can figure out solutions when new situations arise” (p.131) |  |
| Dykes  (2020) | L1 | L2 | L2 | L2 | L2 |
|  | “nurses completed the poster … with the patient and family (if available)” (p.4)  “reviewed with the patient and family at admission and during each shift” (p.4) “patients were included in risk assessment” (p.7) | “developing a personalized prevention plan” (p.2) “tailor prevention plans based on their knowledge of the patient” (p.4) “reviewed with the patient and family at admission and during each shift” (p.4) | “patients are prepared to carry out specific and actionable interventions” (p.7) “link patient-specific risk factors to interventions most likely to prevent a fall” (p.8) | “patients’ nonadherence to their fall-prevention plan” (p.2)  “patients were more likely to follow their prevention plan” (p.7)  “patients are prepared to carry out… interventions” (p.7) |  |
| Patient engagement was classified into three levels: • Level 1 (Passive engagement): Decision-making is clinician-led, and patients receive and accept information  • Level 2 (Shared decision-making): Patients participate in decision-making through interaction with healthcare professionals  • Level 3 (Autonomous decision-making): Patients take a leading role in decision-making and behavioral adjustment | | | | | |

**Supplementary Table S2 (continued)**

| **Author**  **(Year)** | **Risk awareness** | **Co-planning** | **Goal setting** | **Self-management** | **Overall** |
| --- | --- | --- | --- | --- | --- |
| Hill  (2015) | L2 | L2 | L3 | L3 | L3 |
|  | “aimed to alert patients to their personal risk of falls” (p.2593)  “raise their knowledge about falls… and motivate them” (p.2593) | “individually tailored follow-up sessions” (p.2593) “tailored for the patient’s individual circumstances” (p.2593) | “helped the patient to set personal goals” (p.2593) “patients set a total of 1643 behavioural goals” (p.2596) “completed a written action plan” (p.2594, 2596) | “patients to engage in suitable… strategies” (p.2597) “capable and motivated to engage” (p.2597) “initiate safe behaviours… especially when alone” (p.2597) |  |
| Morris  (2024) | L1 | L2 | L2 | L2 | L2 |
|  | “using an evidence-based scripted conversation and educational pamphlet” (p.1) “patients are often unaware of their risk of falls” (p.7) | “helped each patient to develop a goal-orientated action plan” (p.3) “plan was conveyed to the supervising clinician” (p.3) | “goal-orientated action plan” (p.3) “give patients simple goals to reduce their risk of falls” (p.7) | “motivational interviewing” (p.3) “brief conversations throughout the admission” (p.3) |  |
| Guo  (2023) | L3 | L3 | L3 | L3 | L3 |
|  | “patients would independently assess fall risk using the i Engaging form” (p.1117) “instructed the patient to fill in the ‘i Engaging form’… to assess the fall risk” (p.1118)  “provided an autonomous assessment of the risk of falls” (p.1117) | “selected the fall prevention measures that the patient was able to complete” (p.1118) “nurses and patients jointly developed a personalized fall prevention plan” (p.1122) | “select fall prevention measures that could be completed” (p.1117) “patient signed and marked… according to mastery of measures” (p.1119) “patient could choose colour cards… as a reminder” (p.1118) | “patients participated in… implementation and dynamic evaluation” (p.1121) “patient could inform the nurse at any time” (p.1119) “patient performed daily behaviour intervention” (p.1119) |  |
| Radecki  (2020) | L2 | L2 | L2 | L2 | L2 |
|  | “patient-directed fall self-assessment” (p.221) “patients as experts in their own preferences and values” (p.220) “nurse and the patient talked about the identified risk factors” (p.222) | “developed a fall prevention plan together” (p. 222) “patient-coproduced safety plan” (p. 221) “engage patients in the development of their safety plan” (p. 220, 224) | “plan… update as needed” (p.222) “used to amend the safety plan” (p.222) | “patients as safety collaborators” (p.220) “did not significantly affect… adherence to the fall plan” (p.224) |  |
| Patient engagement was classified into three levels: • Level 1 (Passive engagement): Decision-making is clinician-led, and patients receive and accept information  • Level 2 (Shared decision-making): Patients participate in decision-making through interaction with healthcare professionals  • Level 3 (Autonomous decision-making): Patients take a leading role in decision-making and behavioral adjustment | | | | | |

**Supplementary Table S3 Cognitive Eligibility, Measurement Participation, and Family Proxy Roles in Patient-Engaged Fall Prevention Studies**

| **Author (Year)** | **Cognitive eligibility for intervention** | **Cognitive requirements for measurement** | **Family role as proxy / engagement** | **Specific evidence (direct quotes)** |
| --- | --- | --- | --- | --- |
| Dykes (2017) | Not reported | Not reported | Passive (information recipient) | No specific criteria reported for cognitive inclusion; family role described as recipients of information (e.g., “communicate fall risk and a tailored prevention plan”). |
| Duckworth (2019) | Included; proxy allowed for cognitively impaired patients | Not reported | Proxy / essential partner | Patients must be aged ≥18 years; either alert and oriented or have family present and involved in care. |
| Christiansen (2020) | Not explicitly restricted | Restricted to cognitively able participants (survey-based outcomes) | Active in intervention / absent in measurement # | Prior to surveying patients, study staff checked that participants were physically and mentally able to participate. Participants were alert and oriented, spoke English, and consented. |
| Dykes (2020) | Included (all adult inpatients; proxy implicitly allowed) | Not reported | Active partner / surrogate | All adult inpatients were included; patient/family could express fall risk factors and prevention plans. |
| Hill (2015) | Restricted to cognitively intact participants (MMSE >23/30 or AMTS >7/10) | Not separately described | Not specified | Patients required adequate cognitive function (e.g., MMSE >23/30 or AMTS >7/10). |
| Morris (2024) | Restricted (patients with significant cognitive impairment excluded; MMSE <24) | Not separately described | Not specified | Patients with significant cognitive impairment (MMSE <24) were excluded. |
| Guo (2023) | Restricted (no cognitive impairment; clear communication ability required) | Not separately described | Not specified | Inclusion criteria required no cognitive impairment and clear communication ability. |
| Radecki (2020) | Restricted (no cognitive deficits required for PFAT participation) | Not separately described | Proxy not central / not explicitly defined | Patients had to be free of cognitive deficit. |
| # Indicates that patients participated in the intervention but were excluded from outcome measurement due to cognitive requirements. Cognitive eligibility refers to whether cognitive status was used as an inclusion or exclusion criterion for participation in the intervention. Cognitive requirements for measurement refer to whether cognitive or communicative ability was required specifically for outcome assessment (e.g., surveys, interviews), regardless of intervention eligibility. Family roles were categorized as passive (information recipient), proxy (acting on behalf of the patient), or active partner (engaged in shared decision-making or intervention processes). | | | | |
